# The College of Nursing: Some Absurdities and a Warning

**Published:** 1916-06-10

**Authors:** 


### THE MATRONS' AND SISTERS' DEPARTMENT.

### THE COLLEGE OF NURSING.

### SOME ABSURDITIES AND A WARNING.

It is a sign of the times, and supplies a further proof of the want of thought which so many people exhibit at the present time, that before the College is organised, or in a position to start on its way, opposition, or at least a feeling of grievance, is credited to certain organisations which we venture to believe the fairer and clearer minds among the managers of them will declare to be at least exaggerated. The "Council"—clearly the Managers are meant—of the Metropolitan Asylums Board, for instance, is reported in some nursing papers to have supported the grievance in question. We have before us the printed report of the Hospital Committee of this Board, which in effect merely indicates certain points raised by this Committee arising out of its consideration of the letter of the Hon. Arthur Stanley, chairman of the Council of the College of Nursing, a copy of which appeared in The Hospital of April 29, 1916. The Managers constitute a public body with grave responsibilities, and it is only an act of prudence on their part to adopt the Committee's suggestion to invite Mr. Stanley to supply them with further information on the points raised, and meanwhile to reserve a vacancy on the governing body of the College at the disposal of the Managers should they decide to nominate representatives after they have had an opportunity of considering the further information with which they have asked to be supplied. Again, it is definitely stated that "the Fever Nurses' Association is hurt that it was not officially invited to put a representative on the Council," and this feeling is also credited to the Metropolitan Asylums Board, despite the facts just stated, which clearly prove that it was Mr. Stanley's invitation to send two representatives which has caused that body to ask for further information on various points. These statements are in amusing conflict with each other, and seem to indicate, on the part of the nursing papers in which they appear, a willingness to wound the scheme of the College though afraid to strike it directly.

Prejudice is also stated to be at work, and in proof of this the case is quoted of a girl who has had three years' fever training in an excellent Scottish fever hospital who declares that the fact of having fever training has gone against her in her applications to several large London hospitals. A spirit of compromise by the College of Nursing is recommended as a remedy for this, which we should be inclined to think will at once be found if the girl in question will make her application to some of the general hospitals in Scotland, where the authorities will be better able to appraise her merits and that of the fever training she has undergone.

Again, Miss Broadwood and others are already raising questions in regard to the position of the district, village, and cottage nurses. There is a time for everything, and Miss Broadwood and others like her must have patience, for it must be evident that the first thing to do is to deal with certificated trained nurses, and until their case has been met and provided for it will not be possible to deal with a number of smaller though important questions, of which Miss Broadwood's is one.

It is widely recognised throughout the nursing world that the extreme partisans and advocates of State Registration by demanding the Bill, the whole Bill, and nothing but the Bill, have it in their power to break away from agreement and the College of Nursing by refusing all compromise and any modification of the proposals as they stand in the Bill. These extremists are relatively few in number, but amongst them are some who have so committed themselves to one particular policy that they are in imminent danger of becoming fanatics on this question. The success of the British nation and Empire has been the spirit of compromise which is dear to the British people and has resulted in making them exemplars and leaders amongst the nations of the world. We venture to point this out whilst the Conference on Registration is still in session, because we are confident that the profession of nursing and the British electorate and their representatives in the House of Commons will never consent to impose the extreme views of a small section of the nursing world upon the nursing profession as a whole. In this connection it is well to keep this truth in mind, that had the advocates of nurse registration from the earliest days exhibited a spirit of compromise and a sole desire to benefit the nurses by registration, State registration would have been sanctioned by Parliament long ago on the only practical and sufficient basis.

